# Porous Biphasic Calcium Phosphate Granules from Oyster Shell Promote the Differentiation of Induced Pluripotent Stem Cells

**DOI:** 10.3390/ijms22179444

**Published:** 2021-08-31

**Authors:** Wen-Fu Ho, Mei-Hwa Lee, James L. Thomas, Jin-An Li, Shih-Ching Wu, Hsueh-Chuan Hsu, Hung-Yin Lin

**Affiliations:** 1 Department of Chemical and Materials Engineering, National University of Kaohsiung, Kaohsiung 81148, Taiwan; m1075612@mail.nuk.edu.tw; 2 Department of Materials Science and Engineering, I-Shou University, Kaohsiung 84001, Taiwan; meihwalee@ntu.edu.tw; 3 Department of Physics and Astronomy, University of New Mexico, Albuquerque, NM 87131, USA; jthomas@unm.edu; 4 Department of Dental Technology and Materials Science, Central Taiwan University of Science and Technology, Taichung 40601, Taiwan; scwu@ctust.edu.tw (S.-C.W.); hchsu@ctust.edu.tw (H.-C.H.)

**Keywords:** oyster shell, porous hydroxyapatite granules, biphasic CaP bioceramics, induced pluripotent stem cells, biocompatibility

## Abstract

Oyster shells are rich in calcium, and thus, the potential use of waste shells is in the production of calcium phosphate (CaP) minerals for osteopathic biomedical applications, such as scaffolds for bone regeneration. Implanted scaffolds should stimulate the differentiation of induced pluripotent stem cells (iPSCs) into osteoblasts. In this study, oyster shells were used to produce nano-grade hydroxyapatite (HA) powder by the liquid-phase precipitation. Then, biphasic CaP (BCP) bioceramics with two different phase ratios were obtained by the foaming of HA nanopowders and sintering by two different two-stage heat treatment processes. The different sintering conditions yielded differences in structure and morphology of the BCPs, as determined by scanning electron microscopy (SEM), X-ray diffraction (XRD), and Brunauer–Emmett–Teller (BET) surface area analysis. We then set out to determine which of these materials were most biocompatible, by co-culturing with iPSCs and examining the gene expression in molecular pathways involved in self-renewal and differentiation of iPSCs. We found that sintering for a shorter time at higher temperatures gave higher expression levels of markers for proliferation and (early) differentiation of the osteoblast. The differences in biocompatibility may be related to a more hierarchical pore structure (micropores within macropores) obtained with briefer, high-temperature sintering.

## 1. Introduction

Taiwan has a history of oyster breeding on its western coast, producing large quantities of oyster shells that have been abandoned/discarded as a waste product [1]. Oyster shells consist of 91–95% calcium carbonate (by weight) [2], 4 wt% organic matter, and small amounts of oxides [3]. The possible use of oyster shells is to produce calcium phosphates (CaP) for biomedical applications [4,5,6], which has been reviewed by Habraken et al. [7]. Calcium phosphate ceramics exhibit various phases and combinations thereof, though they are mainly used in the form of hydroxyapatite (HA), β-tricalcium phosphate (β-TCP), or biphasic calcium phosphate (BCP) [8]. HA is the main mineral component of human bones and is a common filling material for orthopedics and dentistry owing to its high biocompatibility and nontoxicity. It has recently been used in vital pulp therapy to promote the repair and regeneration of dental pulp tissues [9,10]. It has been used as a tissue engineering scaffold that induces the growth of osteoblast cells [11], for the cellular transfection of genes, and as a drug carrier [12,13,14]. Additionally, HA can be coated on the surfaces of polyamidoamine dendrimers to improve their oxygen-carrying capacity and the resistance of heme to damaging oxidation [15].

Biodegradability is a critical property of artificial bone fillers, which must not hinder the growth of new bone; therefore, the degradation rate has to adapt to (or at least be compatible with) the formation of new bone [16]. To improve performance in medical applications, high-temperature processing is essential for the preparation of apatites for biomaterials [17]. Of the calcium phosphate forms, HA is thought to have the most properties [18], while tetracalcium phosphate and α-tricalcium phosphate (α-TCP) are the most degradable apatites [19], about twice as soluble as sintered HA at physiological conditions (e.g., pH). β-TCP has the same composition as α-TCP but with a different lattice structure, resulting in slightly reduced degradability and favorable bioresorption but poorer bioactivity [20]. Therefore, many clinical bone filling materials are composed of a biphasic combination of HA and β-TCP [21,22]. 

Many apatite filling materials with porous granules have been developed and applied clinically [23]. These materials have an interconnected porous structure, similar to that of natural bone, allowing the even penetration of bone cells throughout the granules. Several techniques have been employed to prepare porous apatite granules, resulting in different porosities, pore distributions, and mechanical properties [23]. Moreover, the bone regeneration potential of human-induced mesenchymal stem cells (iMSCs) has been explored by transplanting calcium phosphate granules with iMSCs [24]. Bone mesenchymal stem cell-enriched β-tricalcium phosphate scaffold has been shown to promote the regeneration of diaphyseal bone [25]. 

Most biological apatites are nonstoichiometric and contain many essential trace elements such as Mg, Mn, Zn, Na, and Sr. Many studies have synthesized calcium phosphate doped with trace elements; for example, the synthesis of Zn-doped BCP fine powders [26] or incorporating various levels of Mn into BCP [27] have been demonstrated via a sol–gel route. Our previous work used oyster shells as a raw material for the synthesis of HA [3,28,29]. The natural biological origin of oyster shells, containing calcium and several trace elements such as Na, Mg, and Sr, may help to produce structures and compositions similar to that of human bone [28]. In the present work, nanograde HA powders were synthesized from oyster shells by liquid-phase precipitation, and then porous HA granules were prepared by the foaming process. BCPs with two-phase ratios were obtained from porous granules under sintering conditions. Their morphology and crystal structure were characterized by scanning electron microscopy (SEM) and X-ray diffraction (XRD), respectively. Finally, their biocompatibility and ability to modify gene expression in iPSCs were examined using cell counting kit 8 (CCK8) and the real-time quantitative reverse transcription-polymerase chain reaction (qRT-PCR) assay. 

## 2. Results and Discussion

**Figure 1:**
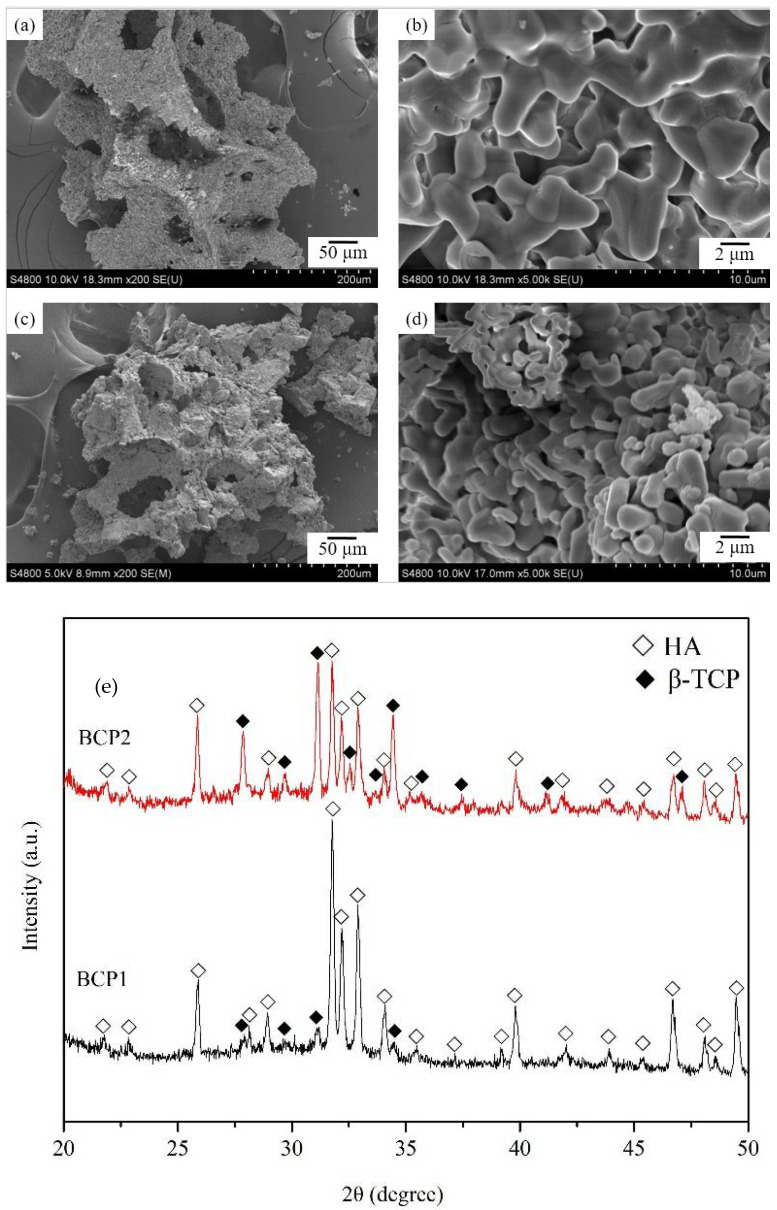
FE-SEM images of BCP1 (**a**,**b**) and BCP2 (**c**,**d**) granules; (**e**) XRD patterns of BCP1 and BCP2 granules.

Figure 1 displays the FE-SEMs of both forms of BCP granules. The low-magnification images (Figure 1a,c) suggest that the granules have interconnected pores. The high-magnification photos (Figure 1b,d) show that many micropores (diameters < 100 μm) formed during the sintering of the powder particles. BCP1, sintered at a higher temperature for a shorter time, was comprised of larger, more rod-shaped particles compared to BCP2. The micropores seen in these materials may provide channels for the transportation of body fluids and nutrients, which are critical for bone regeneration and growth. Figure 1e shows the XRD patterns of BCP1 and BCP2 granules. The Joint Committee on Powder Diffraction Standards (JCPDs) card numbers for HA and β-TCP are 09-432 and 09-169, respectively. The diffraction patterns of BCP1 and BCP2 granules exhibit no peaks from residues of raw materials or products of phase decomposition other than the expected HA and β-TCP phases. Furthermore, the XRD patterns reveal that the composition ratios of the HA and β-TCP phases are around 90:10 and 50:50 for BCP1 and BCP2, respectively.

**Figure 2:**
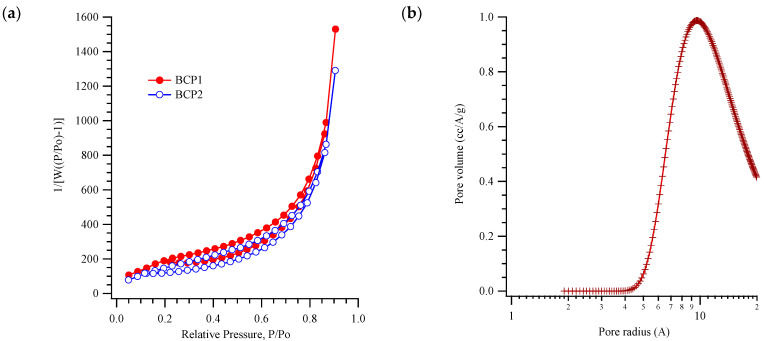

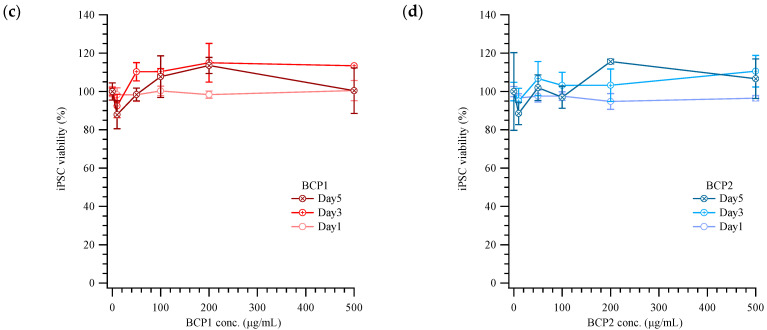
(**a**) The nitrogen adsorption and desorption on BCP1 and BCP2 porous granules; (**b**) pore size distribution of BCP1 and BCP2 fitted by the Barrett–Joyner–Halenda (BJH) model. Viability of iPSCs with various concentrations of (**c**) BCP1 and (**d**) BCP2 on the 1st, 3rd, and 5th day, normalized to unexposed control cells.

**Figure 3:**
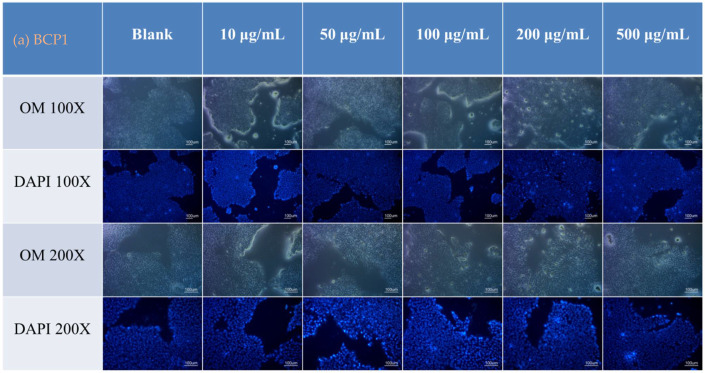

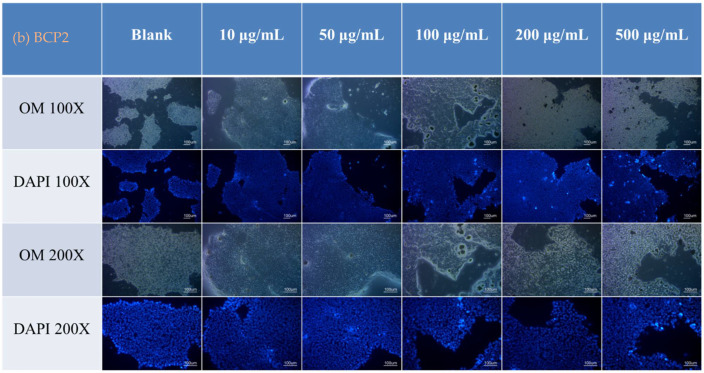
Optical microscopy imaging (OM) and nuclear (DAPI) staining of iPSCs incubated with various concentrations of (**a**) BCP1 and (**b**) BCP2 on the 1st, 3rd, and 5th day.

The nitrogen gas adsorption–desorption curves and pore size distribution curve are shown in Figure 2a,b. The specific surface areas are 7.6 ± 0.4 and 9.1 ± 0.3 cm^2^/g for BCP1 and BCP2, respectively, by the Brunauer–Emmett–Teller (BET) adsorption equation [30]. According to the International Union of Pure and Applied Chemistry (IUPAC) classification of hysteresis loops [31], both BCP1 and BCP2 have type H3 loops; thus, slit-shaped pores are expected for BCPs. The average pore radii are about 3.7 nm and 0.85 nm for BCP1 and BCP2, respectively, calculated by the Horvath–Kawazoe (HK) model, Figure 2b. Notably, these micropores and the rough macropore surfaces are known to be osteoinductive for stem cells. The presence of micropores within macropores (Figure 1) increases the area for protein adsorption, favoring cell differentiation and bone matrix deposition [32]. Figure 2c,d presents the viabilities of iPSCs incubated with BCP1 or BCP2 after 1, 3, and 5 days. Viabilities were normalized to that of control cells not exposed to BCPs. Cells exposed to modest concentrations of BCPs (e.g., 200 µg/mL) grew nearly 20% better than controls, but, interestingly, low BCP1 and BCP2 concentrations (10 µg/mL) were slightly inhibitory. The optical and DAPI staining, shown in Figure 3, also indicates high biocompatibility. Two dosages of BCPs that enhanced the growth of iPSCs (100 and 500 µg/mL) were then employed for the examination of gene expression. 

**Figure 4:**
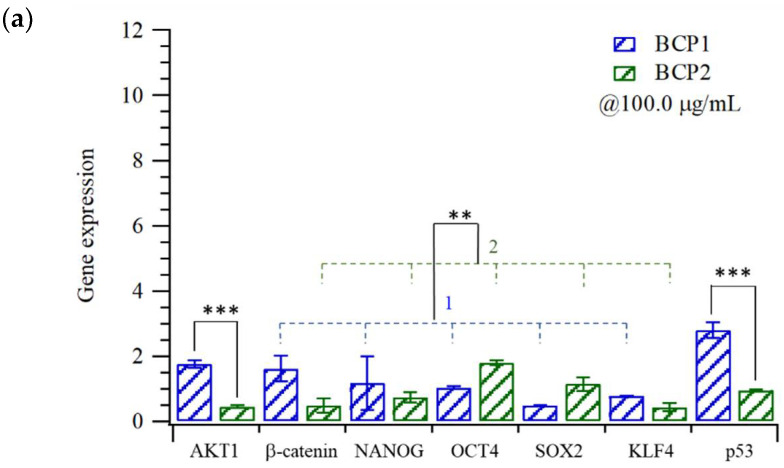

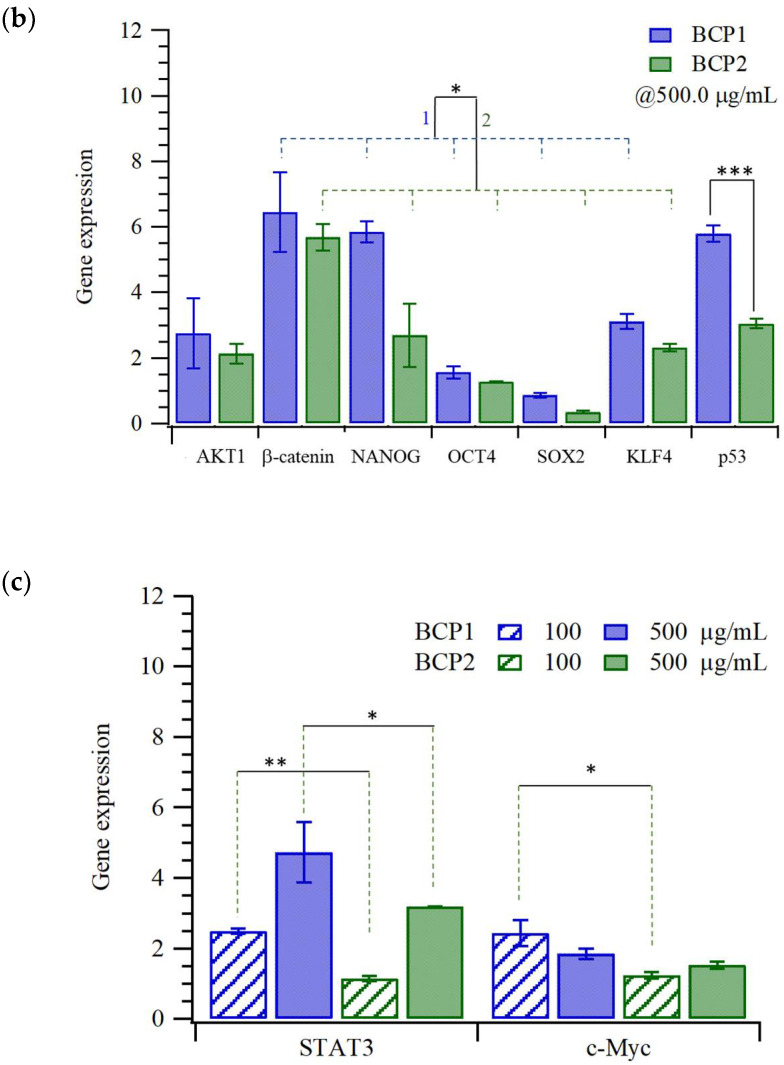
Expression levels (relative to unexposed controls) of key factors in differentiation/self-renewal pathways of iPSC with (**a**) 100 and (**b**) 500 µg/mL of BCPs; (**c**) comparison of the expression of STAT3 and c-Myc of iPSC incubated with 100 and 500 µg/mL of BCPs (* *p* < 0.05; ** *p* < 0.005; *** *p* < 0.0005).

The differentiation of human iPSC-derived mesenchymal stem cells (iMSCs) for bone regeneration has recently been reported [24]. iMSCs can further differentiate into osteoblasts in vitro with the expression of bone morphogenic proteins (BMPs) and secretion of paracrine signaling-associated cytokines. We cultured iPSCs in contact with BCP1 or BCP2 and measured the expression levels of a number of factors in the differentiation pathways, shown in Scheme S1. Gene expression levels, relative to control cells not exposed to BCPs, are shown in Figure 4. High expression of some reprogramming factors (such as NANOG and KLF4) was found in iPSC with BCPs at 500 µg/mL (Figure 4a,b). Notably, the high expression of the p53 gene may reveal a possible gene repair process. p53 expression for BCP2 was around one-third and one-half of that found for BCP1, at 100 and 500 µg/mL dosages, respectively. Figure 4c shows that the expression of SATA3 increases with the BCP concentration but is still lower for BCP2 at both concentrations than for BCP1. The differentiation of these iPSCs seems greater for BCP1 (at low β-TCP), especially at higher concentrations, as seen in Figure 4c. 

**Figure 5:**
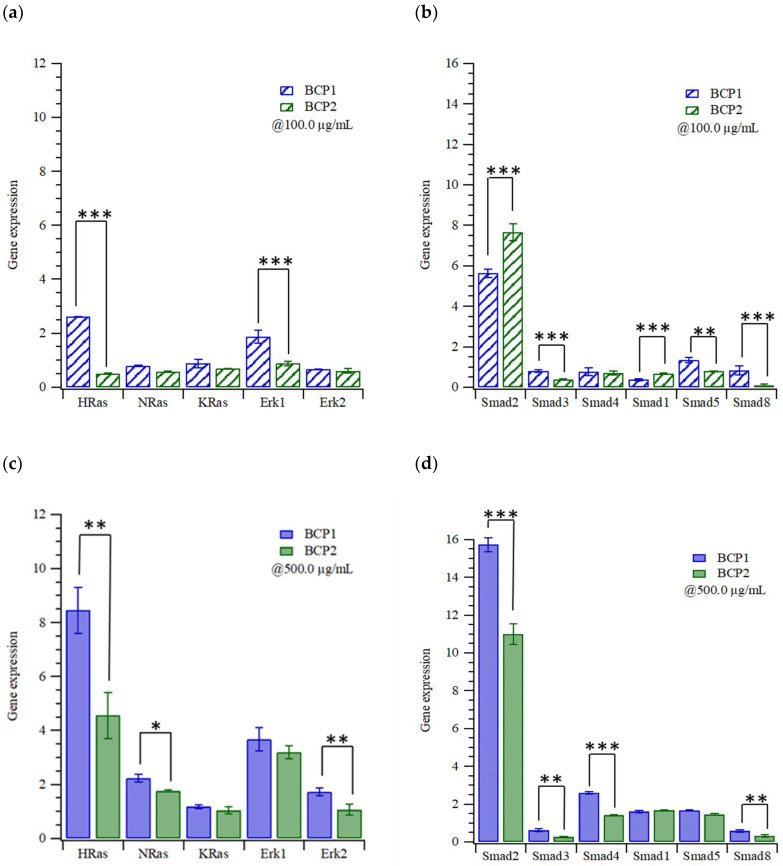
Expression levels of (**a**,**b**) Ras and ERK or (**c**,**d**) Smads in iPSCs exposed to (**a**,**b**) 100 and (**c**,**d**) 500 µg/mL of BCPs (relative to unexposed controls) (* *p* < 0.05; ** *p* < 0.005; *** *p* < 0.0005).

Wu et al. reviewed transforming growth factor beta (TGF-β) and bone morphogenetic protein (BMP) signaling in osteoblast skeletal development, and in bone formation, homeostasis, and disease [33]. Figure 5 shows the TGF-β signaling in iPSCs (Scheme S2) induced by BCPs. Figure 5a,c displays the expressions of Ras (a small GTPase) and extracellular signal-regulated kinase (ERK). BCP1 causes higher expression levels for both markers than does BCP2; their mRNA expression levels with BCP2 at 100 µg/mL are almost the same as those of the controls. The expression of HRas for BCP1 (10% β-TCP) is three times that for BCP2 (50% β-TCP), while ERK1 is double, at 100 µg/mL. As the concentration of BCPs increases, the expression levels for both Ras and ERK dramatically increase. Increasing the expression of ERK promotes the expression of Smads, as shown in Figure 5b,d. The combination of Smad 2/3 with Smad 4 will cross the cytoplasm and bind with coactivator CBP (cAMP response element-binding protein, a histone acetyltransferase), activating other transcription factors [34]. The expressions of other Smad proteins (such as Smads 1/5/8) slightly increased at 500 µg/mL of BCPs in BMP signaling (Scheme S3); this effect may be related to the proliferation and (early) differentiation of the osteoblast [33].

**Figure 6:**
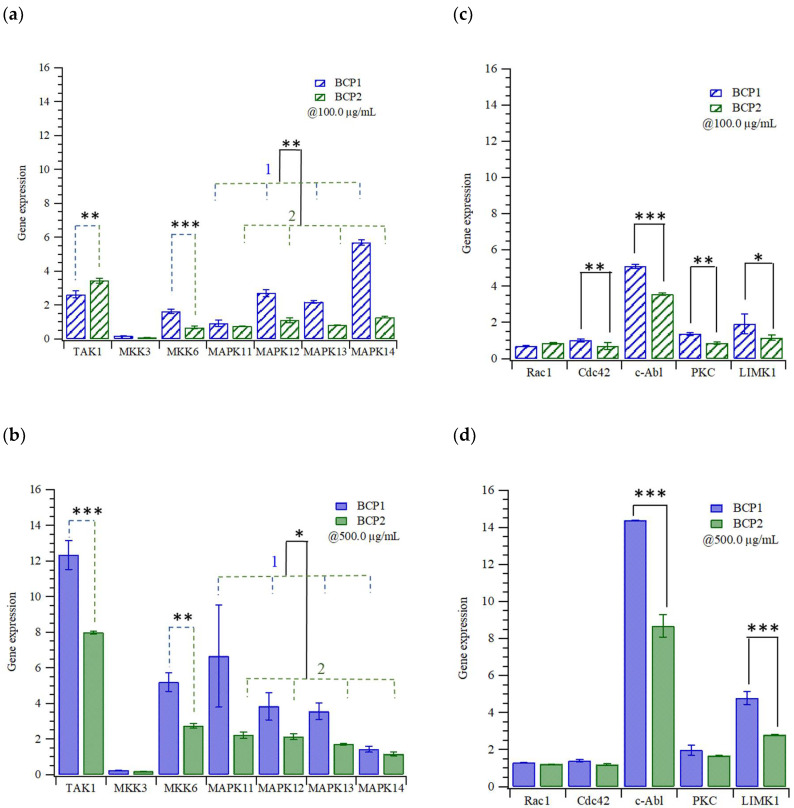
Expression levels in iPSCs with (**a**,**c**) 100 and (**b**,**d**) 500 µg/mL of BCPs for the (**a**,**b**) cellular stress proteins or (**c**,**d**) adhesion/interaction proteins (* *p* < 0.05; ** *p* < 0.005; *** *p* < 0.0005).

In Figure 6a, the expression of Smad proteins also increases the expressions of TGF-β–activated kinase 1 (TAK1) and mitogen-activated protein kinase isoforms 3 and 6 (MKK 3/6) activating p38, revealing that BCPs may generate environmental stresses and inflammatory cytokines [35]. According to Figure 6b, increasing the concentration of BCPs, and especially BCP1 (10% β-TCP), appears to increase the stress on the iPSCs. Finally, the BCPs only slightly increased the expressions of Ras-related C3 botulinum toxin/cell division control protein 42 homolog (Rac/Cdc42), but the expression of the human tyrosine-protein kinase (c-Abl) was very high, as shown in Figure 6c,d. Both c-AbI and protein kinase C (PKC) promote the adhesion of cells, and the expression of LIM domain kinase 1 (LIMK1) may induce actin polymer stress fibers. 

## 3. Materials and Methods

### 3.1. Synthesis of Biphasic Calcium Phosphate (BCP) Porous Granules from Oyster Shells

Oyster shells, collected from Dongshi beach, Taiwan, were carefully washed, crushed, and calcined at 1100 °C for 4 h; then, they were ground to fine powders using an agate mortar and sieved using a 325 mesh screen. The calcined oyster shell powders were added to an aqueous acetic acid solution and stirred with a magnetic stirrer at 320 rpm for 6 h at 55 °C. Next, 0.06 M (NH_4_)_2_HPO_4_ (98 wt%, Showa Chemical, Tokyo, Japan) aqueous solution was added dropwise; aqueous ammonia (NH_4_OH) (28 wt%, Showa Chemical, Tokyo, Japan) was then used to adjust the pH value to 10, and stirring was continued resulting in the precipitation of calcium phosphate in an hour. This precipitate was centrifuged and washed in deionized water and dried in a 70 °C oven for 48 h. Coconut oil (35 wt%, Choneye Pure Chemicals, Taipei, Taiwan) was added (1.5 mL/g) to the above-prepared powders while stirring for 15 min to induce foaming. The foam containing powder was squeezed onto a crucible and dried in a 70 °C oven for 2 h.

The foamed powders were then subjected to a two-stage heat treatment process to obtain porous granules with two different phase ratios (HA/β-TCP), BCP1 and BCP2. The granules were first calcined at 700 °C for 2 h at a heating rate of 2 °C/min and cooled to room temperature in the furnace (CMF-15HS, Cheng Sang, Changhua, Taiwan). To form BCP1, the granules were heated to 1300 °C at a heating rate of 2 °C/min, kept at 1300 °C for 20 min, and then slowly cooled in the furnace. To form BCP2, granules were heated to 1000 °C at a heating rate of 2 °C/min, kept at 1000 °C for 25 h, and then slowly cooled in the furnace.

### 3.2. Characterization of Biphasic Calcium Phosphate (BCP) Porous Granules 

Nitrogen adsorption measurements were performed with a NOVA 1000e, and Brunauer–Emmett–Teller (BET) analysis was performed with the Autosorb program (Quantachrome Instruments, Florida, USA). BET analysis was carried out using nitrogen gas at a relative vapor pressure of 0.05–0.3 at 77 K. The sintered porous granules were observed with a field emission scanning electron microscope (FE-SEM, S-4800, Hitachi, Tokyo, Japan). An X-ray powder diffractometer (XRD, D8 Advance, Bruker, Bremen, Germany) was used for crystalline phase analysis, operating with a Cu Kα radiation generated at 40 kV and 40 mA. The diffraction angles (2θ) of each sample were measured from 20° to 50° at a scan speed of 5°/min and a step size of 0.02°. 

### 3.3. Cytotoxicity and Immunohistochemistry of iPSCs with Biphasic Calcium Phosphate (BCP) Porous Granules 

Induced pluripotent stem cell line (iPSC A18945), 98% Essential 8™ basal medium (A1517001), Essential 8 supplement (A1517001), DMEM/F12+ and GlutaMAX (10565018), Geltrex (A1413301) were purchased from Gibco (Thermo Fisher Scientific, Inc., Waltham, MA, USA); the culture protocol can be found in (https://assets.thermofisher.com/TFS-Assets/LSG/manuals/episomal_hipsc_man.pdf accessed on 31 August 2021). iPSCs were cultured in 98% Essential 8™ basal medium with 2% Essential 8™ supplement (50×), 15 mM 4-(2-hydroxyethyl)-1-piperazineethanesulfonic acid) (HEPES, Sigma, St. Louis, MO, USA), sodium bicarbonate (Sigma St. Louis, MO, USA) 1.743 g/L at 37 °C and 5% CO_2_. For the cytotoxicity experiments about 50,000 iPSCs per well were seeded in 24 well tissue culture polystyrene (TCPS) culture plates and incubated with various concentrations of BCPs. The CCK-8 cell counting kit (Sigma Aldrich, Kumamoto, Japan) was used for assessing the viability of cells after exposure. Briefly, CCK-8, (2-(2-methoxy-4-nitrophenyl)-3-(4-nitrophenyl)-5-(2,4-disulfophenyl)-2H-tetrazolium, monosodium salt) solution (50 µL) was added to each well and incubated for 3 h, and then the optical density was measured at 620 nm with a plate reader (Power Wave HT340, BioTek, Winooski, VT, USA). Cell viability (%) was calculated as a percentage with respect to untreated cells on TCPS. After the optical density measurements, iPSCs were washed with 400 µL Dulbecco’s phosphate-buffered saline (DPBS) in each well. The cells were fixed in 350 µL of 3.7% formaldehyde in PBS (pH 7.4) for 15 min at room temperature. After washes with 350 µL DPBS in each well, cells were stained with the nuclear dye (4′,6-diamidino-2-phenylindole, DAPI, 250 µL of 1 µg/mL; Sigma Aldrich, St. Louis, MO, USA) for 5 min. Finally, the cells were washed with DPBS and examined with an inverted fluorescence microscope (CKX41, Olympus, Melville, NY, USA).

### 3.4. Gene Expression of iPSCs Cultured with Biphasic Calcium Phosphate (BCP) Porous Granules 

The sequences (5′-3′) of primers for GAPDH, OCT4, SOX2, c-Myc, Klf4, Akt1, Akt2, β-catenin, STAT3, NANOG, HRAS, KRAS, NRAS, ERK1, and ERK2 and MKK6, MAPK11, MAPK12, MAPK13, MAPK14, RAC1, CDC42, c-Abl, TAK1, MKK3, SMAD2, SMAD3, SMAD4, SMAD1, SMAD5, SMAD8, LIMK1, PKC are listed in Supplementary Tables S1 and S2. iPSCs were centrifuged at 1000 rpm for 5 min to remove culture medium. The total RNA extraction from iPSCs treated with various concentrations of BCPs was accomplished using the Nucleospin RNA, Mini kit for RNA purification (740955.50 Macherey-Nagel GmbH & Co. KG, Dueren, Germany). Complementary DNA was obtained following a Magic RT Mastermix cDNA synthesis kit (BB-DBU-RT-100, Bio-genesis Technologies, lnc., Taipei, Taiwan). Real-time PCR was then performed with IQ2 SYBR Green Fast qPCR System Master Mix (BB-DBU-006-5, Bio-genesis Technologies, lnc., Taipei, Taiwan) in a StepOne™ Real-Time PCR System (LS4376357, Applied Biosystems, Waltham, MA, USA). Relative gene expression was determined using a ΔΔCq method [36] and normalized to a reference gene (GAPDH) and to control (iPSCs) on TCPS. 

### 3.5. Data Analysis

All experiments were carried out in triplicate, and data are expressed as means ± standard deviation. The gene expression data were analyzed with Student’s *t*-test. Statistical significance was set at a *p*-value of less 0.05 and highly significant as *p* < 0.01.

## 4. Conclusions

**Scheme 1:**
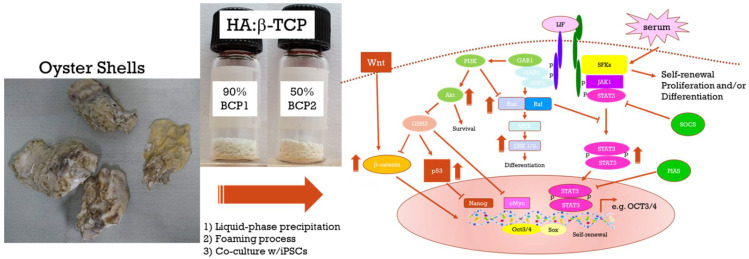
The preparation of BCPs from oyster shells and possible pathways.

This study has provided strong evidence that oyster-shell-derived microporous apatites are suitable to be used successfully for osteopathic, biomedical applications involving hard tissue regeneration. Two materials were synthesized, differing in sintering duration and temperature. BCP1, sintered for a shorter time at a higher temperature, was the more promising of the two: iPSCs cultured with BCP1 particles showed higher expression levels of markers for proliferation and (early) differentiation of the osteoblast [30] than did BCP2. The higher and briefer sintering also results in differences in composition and morphology that may be important in the cellular response. BCP1 particles were larger and more rod-shaped than BCP2 particles and had a much lower fraction of β-TCP phases (HA: β-TCP was 90:10 in BCP1 and 50:50 in BCP2). Moreover, the presence of micropores within macropores especially found in BCP1 likely increases the area for protein adsorption, thus aiding differentiation and bone matrix deposition [29]. BCPs (at 500 µg/mL) increased the expression of reprogramming factors (such as NANOG and KLF4) in iPSCs, but overall, the response to BCP1 was stronger. The preparation of BCPs from oyster shells and possible pathways is depicted in Scheme 1. Taken as a whole, these results strongly encourage future work with oyster-shell-derived biomaterials for biomedical applications.

## Data Availability

The authors confirm that the data supporting the findings of this study are available within the article and its Supplementary Materials.

## Supplementary Materials

The following are available online at https://www.mdpi.com/article/10.3390/ijms22179444/s1.
