# Supplementary material for: Porous Biphasic Calcium Phosphate Granules from Oyster Shell Promote the Differentiation of Induced Pluripotent Stem Cells
Source: Int J Mol Sci. 2021 Aug 31;22(17):9444. doi: 10.3390/ijms22179444 (PMC8430496; doi:10.3390/ijms22179444)
Supplement: Supplementary file 1 [file ijms-22-09444-s001.zip › ijms-1354379-supplementary.pdf]

Supporting inforamtion:

Porous Biphasic Calcium Phosphate Granules from Oyster Shell Promote the Differentiation of Induced Pluripotent Stem Cells

Wen-Fu Ho[1,], Mei-Hwa Lee[2], James L. Thomas[3], Jin-An Li[1], Shih-Ching Wu[4], Hsueh-Chuan Hsu[4], and Hung-Yin Lin[1,*]*

[1]Department of Chemical and Materials Engineering, National University of Kaohsiung, Kaohsiung 81148, Taiwan

[2]Department of Materials Science and Engineering, I-Shou University, Kaohsiung 84001, Taiwan

[3]Department of Physics and Astronomy, University of New Mexico, Albuquerque, NM 87131, USA

[4]Department of Dental Technology and Materials Science, Central Taiwan University of Science and Technology, Taichung 40601, Taiwan

* To whom correspondence should be addressed:
Department of Chemical and Materials Engineering,
National University of Kaohsiung (NUK),
700, Kaohsiung University Rd.,
Nan-Tzu District, Kaohsiung 811, Taiwan
Tel: (O) +886(7)591-9455; (M) +886(912)178-751
E-Mail: linhy@ntu.edu.tw or linhy@caa.columbia.edu

Table S1. The sequence (5'- 3') of primers for GAPDH, OCT4, SOX2, c-Myc, KLF4, Akt1, Akt2, β-catenin, STAT3, NANOG, HRAS, KRAS, NRAS, ERK1, and ERK2.

mRNA	Forward/Reverse	Sequence (5'- 3')
GAPDH	forward	ACAGTTGCCATGTAGACC
	reverse	TTTTTGGTTGAGCACAGG
Akt1	forward	AAGTACTCTTTCCAGACCC
	reverse	TTCTCCAGCTTGAGGTC
β-catenin	forward	CAACTAAACAGGAAGGGATG
	reverse	CACAGGTGACCACATTTATATC
NANOG	forward	CCAGAACCAGAGAATGAAATC
	reverse	TGGTGGTAGGAAGAGTAAAG
OCT4	forward	GACAGGGGGAGGGGAGGAGCTAGG
	reverse	CTTCCCTCCAACCAGTTGCCCCAAAC
SOX2	forward	GGGAAATGGGAGGGGTGCAAAAGAGG
	reverse	TTGCGTGAGTGTGGATGGGATTGGTG
KLF4	forward	ACGATCGTGGCCCCGGAAAAGGACC
	reverse	TGATTGTAGTGCTTTCTGGCTGGGCTCC
STAT3	forward	GGTACATCATGGGCTTTATC
	reverse	TTTGCTGCTTTCACTGAATC
p53	forward	AGGCAGTCAGATCATCTTC
	reverse	TTATCTCTCAGCTCCACG
HRas	forward	ACCATTTTGTGGACGAATAC
	reverse	AAGACTTGGTGTTGTTGATG
KRas	forward	GCCTGCTGAAAATGACTG
	reverse	TCCTGAAGGAATCCTCTATTG
NRas	forward	AGTTTTGTCAGAAAAGAGCC
	reverse	CTAAACTACTGAGAGCTGGG
Erk1	forward	TTCGAACATCAGACCTACTG
	reverse	TAGACATCTCTCATGGCTTC
Erk2	forward	GAAGCATTATCTTGACCAGC
	reverse	TCCATGGCACCTTATTTTTG

Table S2. The sequence (5'- 3') of primers for MKK6, MAPK11, MAPK12, MAPK13, MAPK14, RAC1, CDC42, c-Abl, TAK1, MKK3, SMAD2, SMAD3, SMAD4, SMAD1, SMAD5, SMAD8, LIMK1, and PKC.

Marker	Forward	Reverse
Smad2	AGTGTGTAAAATTCCACCAG	ATTCTAGTTAGCTGATAGACGG
Smad3	CTACCAGAGAGTAGAGACAC	TCTCTGGAATATTGCTCTGG
Smad4	AAAGGTCTTTGATTTGCGTC	CTATTCCACCTACTGATCCTG
Smad1	GGCATATTGGAAAAGGAGTTC	AGATGCTACTGTCACTAAGG
Smad5	CCAGTCTTACCTCCAGTATTAG	TCCTAAACTGAACCAGAAGG
Smad8	CCTATCCTGACTCTTTCCAG	AACTGAGTGTGATAGGGAC
TAK1	CCCAGTGTCAGAATGATTAC	TATCTGTGGAATCATCAGGG
MKK3	CACTATTCAGAGAGGGAGAC	GTTTTTATCCAGCACCTTCC
MKK6	CATCTTGATTCCCTGAAAGTC	CCTTTCGACTGAGACATTTTC
MAPK11	TCAACTGGATGCATTACAAC	GAGGAGATTTTTGCCAGAAC
MAPK12	AAGGAGATCATGAAGGTGAC	GTCAGGATAGAGGCAAAATC
MAPK13	GAAGATTCTGGATTTTGGGC	CTTGAACAGAGTTTTCCCTG
MAPK14	AGATTCTGGATTTTGGACTG	CCACTGACCAAATATCAACTG
Rac1	TTGGTGCTGTAAAATACCTG	GGCATTTTCTCTTCCTCTTC
Cdc42	GAACAAACAGAAGCCTATCAC	TTTAGGCCTTTCTGTGTAAG
c-Abl	CTCAGACGAAGTGGAAAAG	GAGTGAGGCATCTCAGG
PKC	CCAAAGTGTGTGGCAAAG	TCAGACTGGTCTATGTTAGC
LIMK1	TTCCTCAAGGAGGTGAAG	TTGATGTACTCAGTGATGAAG

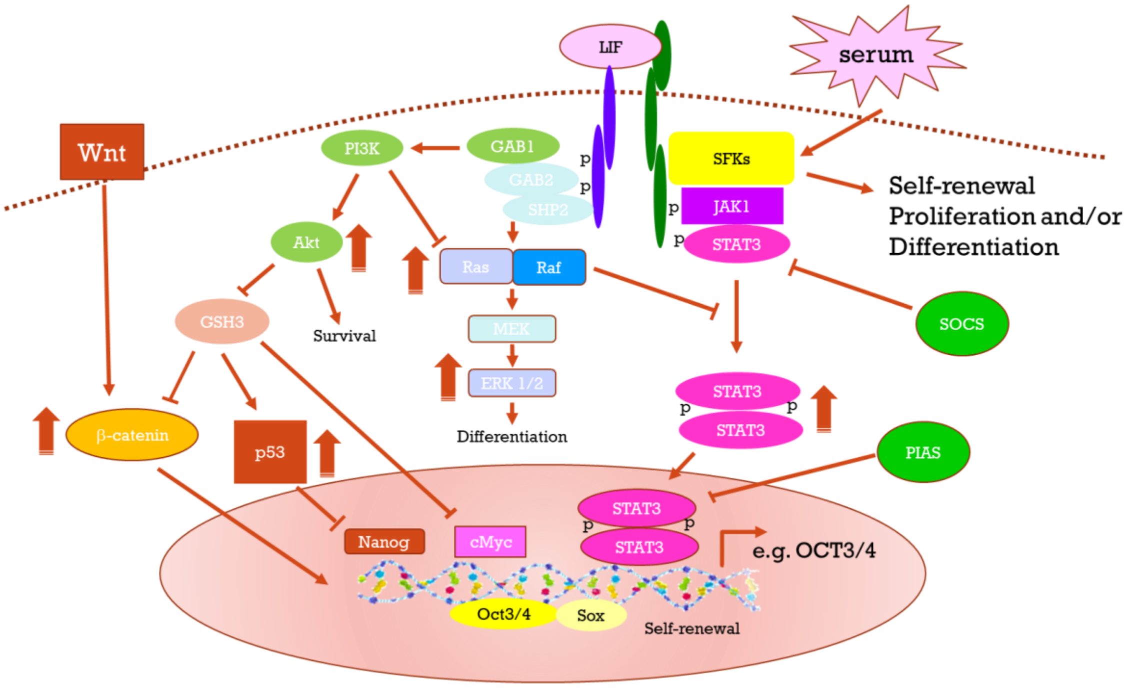

Scheme S1. Signaling of self-renewal or differentiation, and arrows indicate the increase of gene expression with incubation of porous biphasic calcium phosphate granules.

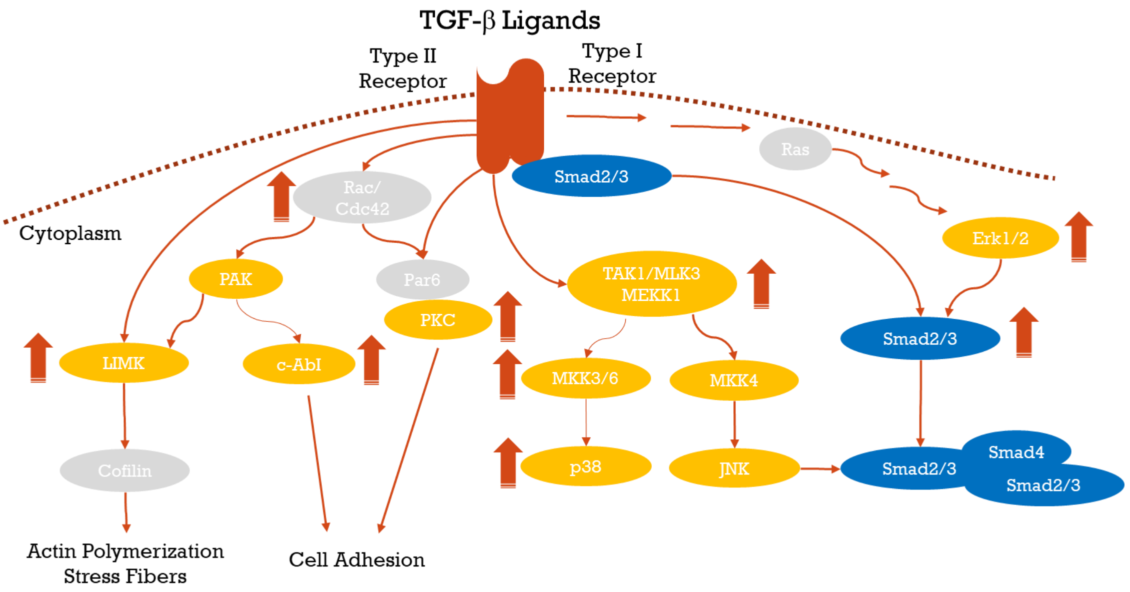

Scheme S2. The transforming growth factor *beta* (TGF-β) signaling in bone [30], and arrows indicate the increase of gene expression with incubation of porous biphasic calcium phosphate granules.

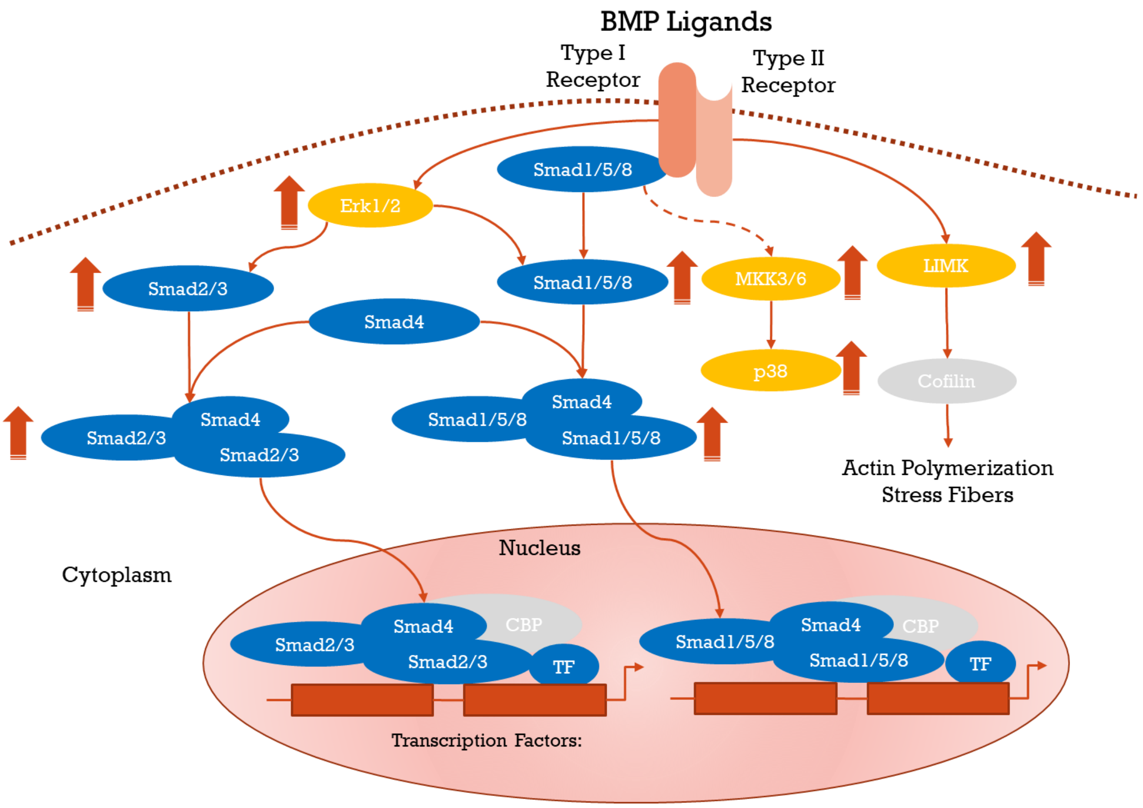

Scheme S3. The bone morphogenetic protein (BMP) signaling in bone [30], and arrows indicate the increase of gene expression with incubation of porous biphasic calcium phosphate granules.
